# Impact of processing steps on cellular content of mechanical isolated stromal vascular fraction

**DOI:** 10.3389/fbioe.2026.1869470

**Published:** 2026-07-17

**Authors:** Marie-Sophie Narzt, Ruth Cardoza, Sandra Milić, Eva Mair, Marlene Wahlmueller, Michael Jakusch, Martin Ganz, Bernard Depypere, Naimeh Hashemi, Andrea Lindenmair, Barbara Schaedl, Clemens Donner, Susanne Suessner, Christopher Kremslehner, Martin Barsch, Heinz Redl, Susanne Wolbank

**Affiliations:** 1 Ludwig Boltzmann Institute for Traumatology, The Research Center in Cooperation with AUVA, Linz/Vienna, Austria; 2 Austrian Cluster for Tissue Regeneration, Vienna, Austria; 3 MorphoMed GmbH, Vienna, Austria; 4 NanoVoxel GmbH, Vienna, Austria; 5 Department of Plastic Surgery, Ghent University Hospital, Ghent, Belgium; 6 University Clinic of Dentistry, Medical University of Vienna, Vienna, Austria; 7 Red Cross Blood Transfusion Service of Upper Austria, Linz, Austria; 8 CDL SKINMAGINE, Department of Dermatology, Medical University of Vienna, Vienna, Austria; 9 Austrian Center for Lipedema, Linz, Austria

**Keywords:** adipose tissue, adipose-derived stromal/stem cells, mechanical isolation, microvascular fragments, processing, regenerative, stromal vascular fraction

## Abstract

Adipose tissue derived stromal vascular fraction (SVF) has emerged as cell therapeutic applicable by point-of-care one-step procedures in autologous settings. Even a mechanical isolation, completed within minutes, is typically followed by multiple steps such as cell washing, filtration, erythrocyte lysis and cryopreservation, which may impact the cell isolate in terms of cell amounts and quality. Using the BioMicroMill, a straightforward device providing mechanically isolated SVF suited for multiple therapeutic doses, we aimed to evaluate the impact of relevant processing steps during isolation and cryopreservation on cell quantity and quality in terms of viability, the presence of regenerative cells, pro-regenerative secretome, vascular network formation. The mechanical isolation yielded in the mean 3.4 × 10^5^ ± 1.42 × 10^5^ SVF cells per ml of lipoaspirate with a mean viability of 38% ± 7.2%. It comprised a heterogeneous mixture of single cells, cell aggregates, extracellular matrix, and microvascular fragments enriched from adipose tissue, providing functionally relevant cell populations (CD31, CD34, CD90, CD105). Adipose-derived stromal cells (ASC) exhibited robust outgrowth, proliferation, and differentiation capacity *in vitro*. The SVF showed pronounced paracrine activity (IL-10, VEGF-A, HGF, IL-6, IL-8, MCP-1), including proangiogenic factors, and supported 3D vascular network formation, demonstrating strong proangiogenic potential. However, all additional SVF processing steps substantially influenced total cell yield, with cumulative cell losses (erylysis 68% ± 16%, filtration 67% ± 16%, washing 54% ± 9%) but no loss of viability or ASC attachment and proliferation *in vitro* was observed. In contrast to other processing steps, filtration substantially altered SVF composition towards single cells, which was also reflected in an altered paracrine activity (significant increase in IL-10 and reduction to minimal levels of HGF). Furthermore, despite further cell loss (mean 47% ± 16%), cryopreservation maintained functional cell populations including ASC, paracrine activity and support of 3D network formation *in vitro*. Taken together, our data demonstrate that processing steps can influence both cell yield and quality. Accordingly, protocol selection should be guided by the intended application and the required functional properties and therefore warrants careful consideration.

## 1 Introduction

Adipose tissue has emerged as a key source in regenerative therapies due to its accessibility, abundance, and rich cellular composition. It comprises a heterogeneous cell population including a mesenchymal stem/stromal cell (MSC) type, the adipose-derived stromal cells (ASC) as well as extracellular matrix (ECM) components. Both, cells and matrix, have evolved as key elements for novel therapeutics in regenerative medicine, attributed to their regenerative, anti-inflammatory, and proangiogenic properties (Bora and Majumdar, 2017; Goncharov et al., 2024).

To harness these regenerative components, various isolation strategies have been developed, broadly categorized into enzymatic and mechanical approaches. Enzymatic digestion, most commonly relying on collagenase, has long been considered the gold standard for isolating the so-called stromal vascular fraction (SVF). This method disrupts adipose tissue architecture, releasing cells of the SVF, including endothelial cells and endothelial progenitor cells, preadipocytes, fibroblasts, erythrocytes, lymphocytes, monocytes/macrophages, pericytes, smooth muscle cells and ASC (Cawthorn et al., 2012; Zimmerlin et al., 2012). These can subsequently be concentrated by centrifugation, which depletes the preparation of mature adipocytes. The final single cell suspension of SVF constitutes a relatively well-defined cell population compatible with common characterizing methods (Bourin et al., 2013). However, enzymatic processing degrades the ECM, alters the native microenvironment and might negatively impacts cell quality (Hendijani, 2017). Moreover, it is associated with higher costs, increased processing time, and regulatory constraints due to its classification as more than minimal manipulation (Trivisonno et al., 2019).

Therefore, mechanical processing protocols and devices based on emulsification, filtration, or cutting have emerged as point-of-care alternatives for both cosmetic and therapeutic applications. These mechanical devices can essentially be divided into two categories for providing SVF: either with the aim of obtaining mechanically fragmented fat—referred to as micro-/nano-fat (Copcu and Oztan, 2020; Dragoo and Chang, 2017; Greenwood et al., 2022; Tonnard et al., 2013; Tremolada et al., 2023), or of isolating SVF cells (Busato et al., 2020). The current study is based on a new closed, sterile and mechanical device (“BioMicroMill”) for fast, simple and straightforward homogenization of adipose tissue through shear forces followed by centrifugation to enrich regenerative cells suitable for the delivery across multiple therapeutic doses. In addition to single cells mechanically isolated SVF (mSVF) in general contains ECM components as well as small and large tissue fragments, particularly microvascular fragments (You et al., 2024).

This composition of mSVF including these fragments represent a closer approximation of physiological tissue conditions and potential enhancement of regenerative capacity.

In clinical applications, even a straightforward mechanical SVF isolation, which is typically completed within few minutes, is usually followed by multiple processing steps including cell centrifugation, filtration and non-enzymatic cell lysis to reduce erythrocyte content, or cryopreservation especially when repetitive dosing is intended. Each of these steps could affect both cell quantity and quality. Depending on the clinical application, the ability to deliver sufficient cell amounts to reach an effective dose (Kim et al., 2026) or to enable repeated administration (Depypere, 2024), may be critical. However, cell quality and identity are equally relevant parameters, which need to be controlled especially in an autologous setting with potentially high donor variability.

Mechanically isolated SVF, the mixture of single and aggregated cells as well as microvascular fragments is particularly susceptible to alterations during processing. Microvascular fragments represent functional vessel segments and have been proposed as vascularization units that promote angiogenesis while preserving ASC and pericytes within their perivascular niche (Ergün et al., 2011). They have demonstrated considerable promise in regenerative approaches across various settings including ischemic wound healing or bone engineering (Gao et al., 2025). From a functional perspective, many applications rely on the proangiogenic potential of SVF and its capacity to deliver regenerative cells to the target site. This proangiogenic contribution may hence be further enhanced by minimizing processing steps, particularly by avoiding excessive manipulation such as filtration.

Within this study, we aimed to systemically evaluate the impact of relevant processing steps during isolation as well as cryopreservation on cell quantity and quality including viability, pro-regenerative secretome and the support for vascular network formation. In addition, ASC were selected as one relevant subpopulation to demonstrate the effects of processing and cryopreservation. To account for variations in cell compositions, we further adapted alternative methods to enable quantitative and qualitative assessment, thereby more accurately reflecting the complexity of the isolate.

## 2 Materials and methods

### 2.1 Adipose tissue

The collection of human adipose tissue lipoaspirate was approved by the local ethical board (JKU Ethical vote 1338/2021) with the participants’ written informed consent. Tissue was harvested during routine outpatient tumescent liposuction procedures performed under local tumescence anesthesia. A vacuum-assisted liposuction device (−70 to −80 kPa) equipped with 4 mm Mirrored tri port II cannulas, was used for all collections to ensure consistent tissue fragmentation and aspiration. Recorded patient information is listed in Table 1.

**TABLE 1:** Donor characteristics recorded under the approved ethical vote (JKU 1338/202).

Parameter	Value
Number of donors (n)	52
Sex	100% female
Age (years)	37.5 ± 12.18 (range 22–67)
BMI (kg/m^2^)	28.6 ± 6.0 (range 20.1–58)
Anatomical site	Abdominal and flanks (standard tumescent liposuction)

Summary of the donor characteristics included in this study (ethical approval JKU, 1338/202).

### 2.2 Sterility testing

Fresh lipoaspirate samples were transported to the laboratory under sterile, temperature-controlled conditions. Upon arrival, for sterility monitoring, 500 µL of lipoaspirate (n = 11), tumescent solution (n = 4) and mSVF supernatant (n = 4) were cultured in duplicates in 2 mL antibiotic-free Dulbecco’s Modified Eagle Medium (DMEM) low glucose, supplemented with GlutaMAX™, sodium pyruvate (Gibco™, Thermo Fisher Scientific Inc., Waltham, Massachusetts, USA) and 10% fetal calf serum (FCS; Corning Inc., Corning, New York, USA). Samples were incubated at 37 °C and 5% CO_2_ up to 7 days and monitored for signs of microbial contamination by visual inspection and microscopy.

### 2.3 Mechanical isolation using BioMicroMill (BMM) device

A mechanical isolation approach was used to obtain mSVF while preserving native ECM components and tissue fragments. All isolations were performed using the BMM mechanical processing system (patent pending), which applies controlled shear and grinding forces to dissociate adipose tissue without enzymatic digestion. For each isolation, 50 mL of lipoaspirate was diluted 1 + 1 with sterile electrolyte solution (ELOMEL (FS-Medizintechnik Handels GmbH, Hallein, Austria)) in a sterile blood bag (MacoPharma S.A., Mouvaux, France) and introduced into the BMM processing unit with the assistance of a controllable pump system. After processing (processing time: 50 s/50 mL lipoaspirate), the resulting suspension was transferred into two 50 mL tubes and centrifuged at 1,000 × *g* for 5 min to separate the mSVF pellet from the lipid and aqueous phases. The lipid layer and supernatant including the floating adipose tissue were removed, and the mSVF pellet was resuspended in 5 mL of endothelial growth medium (EGM-2; Lonza, Basel, Switzerland), DMEM + 10% FCS + 1% Pen/Strep or in cryoSFM media (Gibco™, Thermo Fisher Scientific Inc.).

### 2.4 Enzymatic isolation

For each isolation, 50 mL liposuction material was washed with an equal volume of phosphate buffered saline (PBS), to remove blood and tumescence solution. Lipoaspirate was digested with 0.2 U/mL collagenase NB4 (SERVA Electrophoresis GmbH, Heidelberg, Germany) dissolved in 100 mL PBS containing Ca^2+^/Mg^2+^ and 25 mM N-(2-hydroxyethyl)piperazine-N′-(2-ethanesulfonic acid) (HEPES; Sigma-Aldrich, St. Louis, Missouri, USA), resulting in a final collagenase concentration of 0.1 U/mL at 37 °C under moderate shaking (180 rpm) for 1 h as described previously (Nürnberger et al., 2019). The digested tissue was transferred into 50 mL tubes, centrifuged at 1,200 × *g* for 7 min, following an erylysis step where the cell pellet was incubated with 100 mL erythrocyte lysis buffer (154 mM ammonium chloride (Sigma-Aldrich), 10 mM potassium bicarbonate (Sigma-Aldrich), 0.1 mM ethylenediamine-tetraacetic acid (EDTA; Biochrom, Bernried, Germany) in aqua dest.) for 5 min at 37 °C. The supernatant was aspirated after centrifugation for 5 min at 500 × *g* and the pellet was washed with PBS and filtered through a 100 μm cell strainer (Greiner GmbH, Kremsmünster, Austria). After another centrifugation step at 500 × *g* for 5 min, the SVF cell pellet was resuspended in 10 mL EGM-2.

### 2.5 Histological staining

For histology, adipose tissue samples were fixed overnight in 4% buffered formaldehyde (Carl Roth GmbH & Co. KG, Karlsruhe, Germany), whereas mSVF samples were fixed for 10 min and subsequently embedded in Histogel (Thermo Fisher Scientific). All samples were then rinsed, dehydrated in a graded series of ethanol and stored in 70% ethanol until further processing. Samples were then embedded in paraffin *via* the intermedium xylene (Carl Roth GmbH & Co. KG) and cut in 4 µm thin sections. Sections were histochemically stained with Martius Scarlet Blue (fibrin, collagen, erythrocytes) and hematoxylin and eosin (H&E) to detect structural composition. Samples were analyzed using an Olympus IX85 microscope equipped with CellSense software. Images were acquired at ×4 magnification for an overview and ×10 magnification for detailed analysis.

### 2.6 Cell yield, viability

Freshly isolated SVF samples were processed immediately for cell count and viability assessment using the NucleoCounter® NC3000™ (ChemoMetec A/S, Allerød, Denmark) following the manufacturer’s protocol “Viability and Cell Count using NC-Slide A2™ – Aggregated Mammalian Cells”. In brief, cell count and viability were assessed by measuring two samples in parallel; one treated with cell lysis solution and DAPI to stain all cell nuclei and one without lysis to stain only non-viable cells (cells with membrane damage). Cell yield and viability for cultured cells were quantified by automated cell counting using the “Viability and Cell Count Assay” (NucleoCounter® NC-200™; ChemoMetec A/S). In brief, sample was transferred into a Via-1 Cassette™ which is coated with AcridinOrange to label all cells and DAPI to stain only non-viable cells. Cell number and viability were reported either as absolute values or, for processed samples, as the percentage of cells lost relative to their paired unprocessed control. In parallel, cell viability was also expressed relative to the corresponding paired unprocessed control.

### 2.7 Live staining with calcein AM

Freshly isolated mSVF, the non-adherent fraction of 48 h 2D-cultures and thawed cryopreserved mSVF samples were centrifuged at 300 × *g* for 3 min, resuspended in HBSS buffered- Calcein AM solution (10 µM final concentration; Thermo Fisher Scientific/Invitrogen), and incubated for 30 min at room temperature in the dark. Hoechst 33342 (ChemoMetec A/S) was added prior to analysis (final conc. 2.5 μg/mL), and samples were immediately analyzed by fluorescence microscopy (10×; Olympus IX85 equipped with CellSense software).

### 2.8 ASC cultures

To obtain adherent cells including the ASC, SVF was seeded at a density of 1 × 10^4^ cells/cm^2^ on cell culture plastic in EGM-2 medium and cultured at 37 °C and 5% CO_2_. The first medium change was performed after 48 h, followed by medium changes every 2–3 days. Representative images were acquired on day 2 (d2), 4 (d4) and 7 (d7) using phase-contrast microscopy with an Olympus IX85 microscope at ×10 magnification. On day 7, they were detached, using 1× trypsin/EDTA (Lonza Group AG) at 37 °C, and the cell number was determined. Cell numbers were presented either as absolute values or, in experiments where SVF was processed prior to culture, as relative values normalized to the representative unprocessed controls.

### 2.9 Flow cytometry

ASCs (P0) were centrifuged and washed with Cell wash buffer (BD, Franklin Lakes, NJ, USA) by centrifugation at 500 × *g* for 4 min. The following fluorophore-conjugated antibodies were used to stain the cells in PBS containing 1% BSA for 30 min at 4 °C in the dark: V500-conjugated anti-human CD45 (560777 BD), FITC-conjugated anti-human CD31 (555,445; BD), FITC-conjugated anti-human CD73 (561254; BD), APC-conjugated anti-human CD90 (561,971; BD Bioscience) and PE-conjugated anti-human CD105 (12-1057-42; eBioscience). Following staining, cells were washed with Cell Wash buffer by centrifugation at 500 × *g* for 4 min and immediately analyzed by flow cytometry. Data acquisition was performed on a BD FACSCanto II flow cytometer and analyzed using BD FACSDiva Software. Cell populations were initially identified based on forward (FSC) and side scatter (SSC) characteristics to exclude debris. Doublets were excluded by sequential gating on FSC height *versus* FSC width and SSC height *versus* SSC width parameters. Analysis was restricted to 50.000 singlet cells within the defined FSC/SSC gate.

### 2.10 Differentiation

ASC (P0) were seeded in 12-well plates at densities of 1 × 10^4^ cells/cm^2^ for adipogenic and 5 × 10^3^ cells/cm^2^ for osteogenic differentiation in EGM-2 medium and incubated for 24 h. The following day, the medium was replaced with the respective differentiation medium (StemPro™ Adipogenesis or Osteogenesis Differentiation Kits (Gibco, Thermo Fisher Scientific) according to the manufacturer’s instructions) or EGM-2 as control, and media were subsequently changed every 3–4 days.

On day 14, adipogenic differentiation was assessed by Oil Red O staining. Cells were washed with PBS, fixed with 4% buffered for 1 h, rinsed with water and 70% ethanol, and stained with Oil Red O for 5–15 min. After washing, cells were counterstained with hematoxylin for 1–3 min, rinsed with tap water, and evaluated by light microscopy.

After 21 days, osteogenic differentiation was analyzed with Alizarin Red staining. The cells were washed with PBS and fixed for 1 h with 70% ethanol at −20 °C. After rinsing the fixed cells with aqua dest., the cells were stained with 40 mM Alizarin Red solution (pH 4.2; Merck) for 15 min. The cells were washed with PBS and representative images were acquired using a light microscope.

### 2.11 mSVF processing by erylysis and filtration

mSVF samples were processed by erythrocyte lysis, filtration or washing (sham control). For erythrocyte lysis, cells were resuspended in 25 mL prewarmed lysis buffer to remove erythrocytes, and centrifuged for 5 min at 500 × *g*. For sham control, cells were instead resuspended in prewarmed PBS and centrifuged under identical conditions. For filtration cells were passed through a 100 μm cell strainer (Greiner GmbH) using 25 mL PBS, followed by centrifugation for 5 min 500 × *g*. In all cases, the supernatant was removed, and the cell pellet was resuspended in EGM-2 medium. To assess cell loss during processing, cell counts using NucleoCounter® NC3000™ were performed for each individual sample before and after processing. The percentage of cell loss and the relative change in viability was subsequently calculated. For supernatant harvesting after 48 h and proliferation assays, cells were seeded in 6-well plates at a live cell density of 1 × 10^4^ cells/cm^2^ or cryopreserved in 1 mL CryoSFM (PromoCell, Heidelberg, Germany) with 1 × 10 ^6^ live cells.

### 2.12 mSVF cryopreservation

For comparison of cryopreservation media, mSVF samples were resuspended in either EGM-2 supplemented with 10% DMSO (WAK-Chemie Medical GmbH, Steinbach/Taunus, Germany), DMEM supplemented with 10% FCS and 10% DMSO or CryoSFM. Cryovials containing 1 × 10^6^ viable cells were placed in a controlled-rate freezing container, cooled to −80 °C and transferred to liquid nitrogen after 24 h. Samples were stored for 14–28 days prior to thawing and subsequent analysis. After thawing in a 37 °C warm water bath, nine volumes of DMEM supplemented with 10% FCS were added to the samples followed by centrifugation at 500 × *g* for 5 min to remove residual cryoprotectant and resuspended in EGM-2 medium prior to downstream analyses. To assess cell loss during cryopreservation, cell counts and viability were determined using NucleoCounter® NC3000™ for each individual sample immediately before freezing and directly after thawing, without intermediate processing steps.

To investigate the direct effect of DMSO on cell proliferation across different cryopreservation media (EGM-2, DMEM supplemented with 10% FCS, and CryoSFM), thawed mSVF cells were seeded directly in 6-well plates at a density of 1 × 10^4^ viable cells/cm^2^ in cryopreservation medium diluted with nine volumes of EGM-2 and cultured for 24 h. Subsequently, the medium was replaced with fresh EGM-2. ASC proliferation was assessed after 7 days and calculated relative to freshly isolated mSVF and ASC derived from thawed mSVF, for which DMSO had been removed by washing and centrifugation prior to cultivation.

### 2.13 Secretome analysis by multiplex protein analysis

mSVF cell culture supernatants from freshly mSVF cells (resuspended in DMEM supplemented with 10% FCS and 1% Pen/Strep or EGM-2), processed mSVF and cryopreserved mSVF, all cultured in EGM-2 were seeded at a density of 1 × 10^4^ cells/cm^2^ with 0.232 mL/cm^2^ media After 48 h conditioned media was collected, centrifuged at 1,000 × *g*, for 15 min at 4 °C and stored at −80 °C for further analysis. Samples in two concentrations and the media controls for EGM-2 and DMEM were analyzed by multiplexing for selected factors: Brain-Derived Neurotrophic Factor (BDNF), Hepatocyte Growth Factor (HGF), Interleukin (IL)-1b, IL-10, IL-6, IL-8, Monocyte Chemoattractant Protein (MCP)-1, Nerve Growth Factor (NGF)-b, Platelet Derived Growth Factor (PDGF)-BB, Tumor Necrosis Factor (TNF)-a and Vascular Endothelial Growth Factor (VEGF)-A by the Luminex xMAP technology using the Bio-Plex 200 System (Bio-Rad Laboratories Inc., Hercules, California, USA). Background signals from media controls were subtracted from the corresponding sample prior to data analysis and visualization.

### 2.14 Vascular network formation and proangiogenic support for vascular network formation

To assess the vascularization potential of mSVF cells and their proangiogenic behavior in supporting vascular network formation, 3D fibrin clot cultures were performed (Pill et al., 2015). For mSVF monocultures, 1 × 10^6^ mSVF cells were mixed with fibrinogen (Baxter Healthcare GmbH, Vienna, Austria; 2.3 mg/mL) and thrombin (Baxter, 0.2 U/mL), pipetted onto 18 mm coverslips in 12-well plates and polymerized at 37 °C for 30 min. For SVF–HUVEC cocultures, 0.5 × 10^6^ mSVF cells (fresh and cryopreserved) or 0.5 × 10^6^ fresh eSVF cells were combined with 125.000 HUVEC-YFP and mixed with fibrinogen and thrombin as described above. All clots were cultured for 2 weeks in EGM-2 containing aprotinin (Sigma-Adrich, 100 KIU/mL), with medium changes every 2–3 days. At the endpoint, clots were harvested and processed directly in the 12-well plates.

After cultivation, monoculture and coculture fibrin clots were washed with PBS and incubated with fluorophore-conjugated antibodies: monocultures with AF488 conjugated anti-human CD31 (303110, BioLegend; 1:150 in PBS/1% BSA) and APC conjugated anti-human CD90 (561971; BD Bioscience; 1:100 on PBS/1% BSA), cocultures with AF647 conjugated anti-human CD31 (561654; BD Bioscience; 1:150 in PBS/1% BSA) for 1 h at 4 °C in the dark. Following antibody incubation, Hoechst 33342 (final conc. 2.5 µg/mL) was added and samples incubated for an additional 20 min at 4 °C in the dark. Clots were then washed with PBS, fixed with 3.7% formaldehyde, washed three times, and stored in PBS containing 1% BSA until imaging. Confocal images were acquired on a ZEISS LSM 900 microscope at ×10 and ×20 magnification and analyzed with ZEN software (Carl Zeiss AG, Oberkochen, Germany).

### 2.15 Immunofluorescence staining

The following fluorophore-conjugated antibodies were used for immunofluorescence staining diluted in PBS containing 1% BSA: PE-conjugated anti-human CD45 (555483; BD Bioscience; 1:100), AF488-conjugated anti-human CD31 (303110, BioLegend; 1:150), APC-conjugated anti-human CD34 (555,824; BD Bioscience; 1:100), APC-conjugated anti-human CD90 (561971; BD Bioscience; 1:100) and PE-conjugated anti-human CD105 (12-1057-42; eBioscience; 1:100), with Hoechst as a counterstain.

For 3D analysis of cellular mSVF composition, 0.5 × 10^6^ freshly isolated mSVF cells were incubated with DNase (20 KU/mL; Thermo Fisher Scientific/Invitrogen) in HBSS for 10 min at room temperature prior to embedding in fibrin hydrogels in 12-well plates. After polymerization, hydrogels were incubated with antibody solution (CD45/CD31/CD34; CD45/CD31/CD90) for 30 min at 4 °C in the dark.

Filter residues from mSVF retained in a 100 μm cell strainer during filtration were collected and resuspended in 0.5 mL HBSS containing DNase (20 KU/mL) and incubated for 10 min at room temperature. Filter residues and cells that passed through the cell strainer were centrifuged at 300 × *g* for 3 min and subsequently resuspended in antibody solution (Filter residues: CD31/CD34; CD31/CD90/CD105 and filtered cells: CD31/CD34/CD45; CD90/CD105) and incubated for 30 min at 4 °C in the dark.

After staining, hydrogels, filter remains and filtered cells were washed two to three times with PBS and fixed with 3.7% buffered formaldehyde for 10 min, followed by three to five washing steps and stored in PBS containing 1% BSA until imaging. Filtered cells were spun onto a glass slide at 550 rpm, 5 min with the Cytospin3 device. Imaging was performed using a ZEISS LSM 900 confocal microscope at ×10 and ×20 magnification and analyzed with ZEN software.

### 2.16 Statistical analysis

Statistical analysis was performed using GraphPad Prism 9.5.1 (GraphPad Software, San Diego, California, USA). Paired comparisons were evaluated using a paired t-test, whereas for multiple group comparisons one-way ANOVA with Tukey’s correction for multiple comparisons was performed. Differences in donor variances were tested by F-test. Statistical significance was set at p < 0.05 (*p < 0.05 and **p < 0.01). Results are shown with their mean and standard deviations (SD). The number of biological replicates (n) for each experiment is reported in the corresponding figure legends.

## 3 Results

### 3.1 BMM is suited to isolate mSVF

**FIGURE 1:**
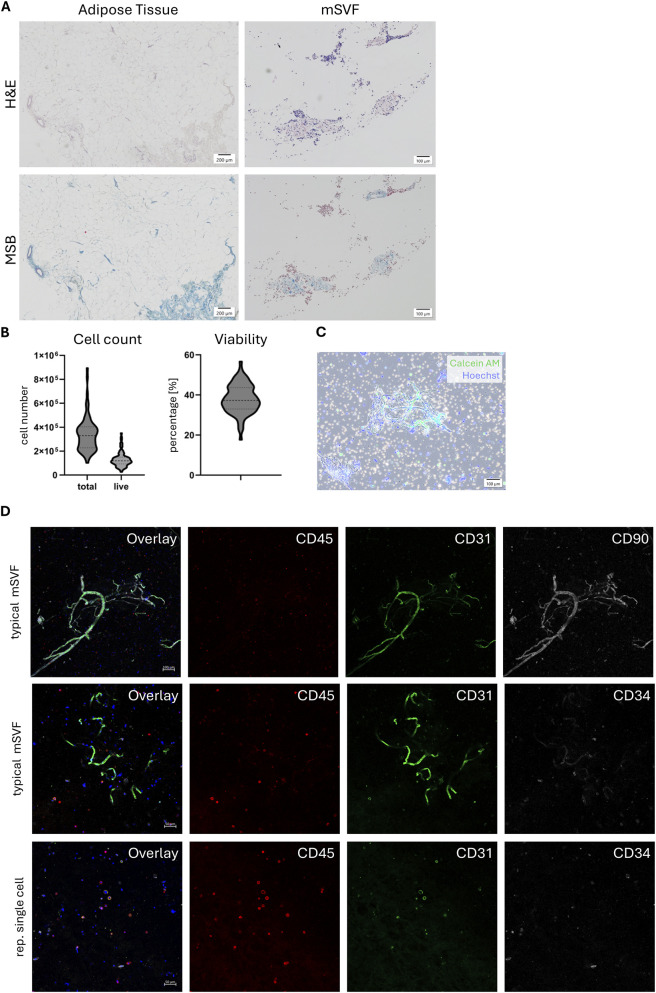
Characterization of freshly isolated mSVF. mSVF was mechanically isolated from adipose tissue **(A)** Representative microscope images of H&E and MSB staining of adipose tissue (scale bar = 200 µm) and the corresponding mSVF isolates (scale bar = 100 µm). **(B)** Violin plots illustrate the distribution of total cell number, live cell number per ml lipoaspirate and viability (n = 77 independent isolations including 48 donors) quantified using an automated cell counter. **(C)** Fluorescence microscopy of freshly isolated mSVF aggregates stained with Calcein AM (green) and Hoechst (blue) with phase contrast overlay; scale bar = 100 µm. **(D)** Representative immunofluorescent staining of freshly isolated mSVF embedded in a three-dimensional fibrin matrix. Upper and middle panel display typical mixed mSVF of single cells and aggregates and lower panel representing only single cell, showing CD45-PE (red), CD31-AF488 (green), and CD34-APC (white) or CD90-APC (white) with Hoechst counterstain, presented as overlays and individual channels; scale bar = 50 μm and 100 µm. Quantitative analyses were performed on n = 77 independent mSVF isolations.

A mechanical isolation system was used to obtain mSVF from lipoaspirate through a rapid (less than 10 min from lipoaspirate to isolated cell pellet), gentle, and fully closed and sterile process. As shown in Figure 1A, mature adipocytes were effectively excluded from the SVF product compared to adipose tissue fragments present in the lipoaspirate, while preserving a highly dense, heterogeneous cell population along with rich ECM as visualized by Martius Scarlet Blue (MSB) staining with partly maintained structural integrity during mechanical isolation.

The total yield of mSVF single cells ranged from 2 × 10^5^ to 4 × 10^5^ cells (mean 3.4 × 10^5^ ± 1.42 × 10^5^) per processed ml of lipoaspirate. This corresponded to a mean live cell number of 1.3 × 10^5^ ± 6.20 × 10^4^ per ml processed lipoaspirate with an average viability of 38% ± 7.2% (n = 77 from 48 donors), as shown in Figure 1B. These results reflect reproducible performance across samples processed under identical conditions.

To complement single-cell quantification, viability was assessed using Calcein AM and Hoechst staining. Combined fluorescence and phase-contrast microscopy revealed the presence of erythrocytes (Hoechst negative), as well as numerous single cells exhibiting strong Calcein AM fluorescence, indicating metabolic activity and viability. In addition to single viable cells, large multicellular structures embedded within ECM components were observed. These structures showed robust Calcein AM fluorescence, confirming their viability (Figure 1C).

To assess the native cellular composition, freshly isolated samples were embedded in a three-dimensional fibrin matrix prior to immunostaining for typical regenerative cell surface markers/marker panel (CD90, CD31 and CD34). This approach minimized cell loss during staining and washing steps and allowed visualization of both, single cells and multicellular structures associated with ECM components, which make up approximately 50% of the mSVF, compared to standard methods like flow cytometry, which requires a single cell suspension. The heterogeneous cell mixture contained CD45-positive (hematopoietic) single cells, indicating the presence of hematopoietic-derived cells. Furthermore, single cells positive for the regenerative cellular spectrum CD90, CD31, or CD34 were observed, indicating the presence of regenerative cell populations within the cell product. The single cells comprise a CD31/CD34 double positive (endothelial cell) population. In addition to individual cells, the mSVF also contained vascular-like multicellular structures exhibiting CD31, CD34 and CD90 positivity, suggesting the presence of preserved microvascular fragments (Figure 1D; Supplementary Figure S1, three-dimensional image).

These findings further confirm that the mSVF retains a heterogeneous and characteristic cellular composition, demonstrated by endothelial, stromal, progenitor, and hematopoietic cell markers.

Sterility was evaluated in samples from eight donors collected at multiple stages of the isolation procedure. All samples were visually monitored for microbial growth throughout the incubation period. No contamination was detected in any samples, demonstrating that both the liposuction procedure and the BMM SVF isolation were performed under sterile conditions supporting the safety of the workflow for potential clinical application (Supplementary Figure S2).

### 3.2 The SVF populations show functional pro-regenerative properties

ASC are a crucial component of the regenerative cell pool in the SVF. According to IFATS and ISCT (Bourin et al., 2013) minimal criteria for MSCs, these cells readily adhere to plastic under standard culture conditions and demonstrate proliferative capacity. To evaluate the presence and fitness of ASC, freshly isolated mSVF cell suspension was plated in 2D cell culture conditions.

**FIGURE 2:**
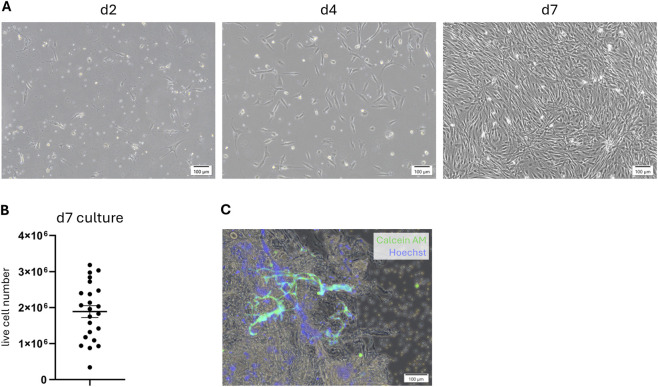
Proliferative capacity and viability assessment of cultured mSVF. **(A)** Representative phase-contrast images showing the plastic adherent cells of cultured mSVF on day 2 (d2), following ASC proliferation on day 4, and day 7. Scale bar = 100 µm. **(B)** Live cell number quantified using an automated cell counter on day 7 shown as mean ± SD (n = 23). **(C)** Representative fluorescence microscopy image of mSVF (d2) stained with Calcein AM (green) and Hoechst (blue) with phase contrast overlay; scale bar = 100 µm. Quantitative analyses were performed on n = 23 independent mSVF cultures.

Adherent cells were observed without the need for an outgrowth culture from tissue fragments. Instead, single cells rapidly attached to the plastic surface and initiated proliferation, with spindle shape-like morphology, as shown in Figure 2A.

By day 7, the number of adherent ASC was quantified, revealing that from 1 × 10^6^ initially plated live mSVF cells, a mean of 1.89 × 10^6^ ± 7.91× 10^5^ ASC were obtained (n = 23) (Figure 2B).

Notably, no endothelial cobblestone-like colonies were observed at early stages of cultures, which are typically obtained among the first adherent cells following standard enzymatic single-cell isolation procedures (Supplementary Figure S3A).

In a comprehensive analysis of five donors, the number of proliferating ASC obtained from 1 × 10^6^ viable SVF cells was comparable between enzymatic and mechanical isolation methods. After 7 days of culture, mSVF yielded a mean cell number of 1.82 × 10^6^ ± 8.48 × 10^5^ cells, whereas SVF isolated using standard enzymatic protocols reached a mean of 1.68 × 10^6^ ± 6.02 × 10^5^ cells, indicating similar (not significant) expansion of adherent ASC from the initial SVF population (see Supplementary Figure S3B).

Furthermore, culture-adapted cells (day 7) derived from both isolation methods exhibited stem cell potential (Supplementary Figure S3D) demonstrated by adipogenic and osteogenic differentiation under induction conditions and expression of typical MSC marker profiles (Supplementary Figure S3C). Collectively, these findings indicate that mSVF contains functionally competent ASC that meet key criteria for mesenchymal stromal/stem cells, including plastic adherence, proliferation, and multilineage differentiation capacity.

In culture, microvascular fragments within ECM components did not adhere to culture plastic but remained viable, as demonstrated by Calcein AM staining with Hoechst counterstaining at day 2 (Figure 2C). These structures formed viable, interconnected networks, indicating preserved cellular integrity and metabolic activity despite the absence of plastic adherence.

**FIGURE 3:**
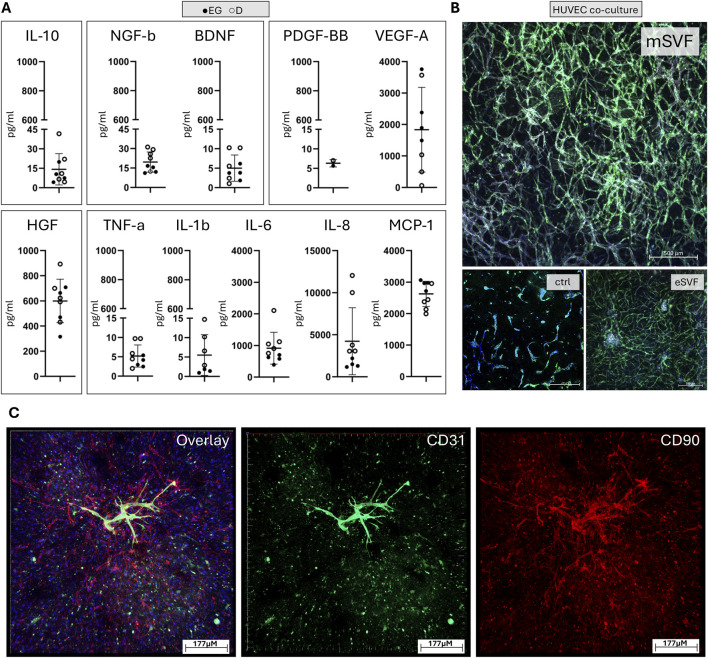
Paracrine signaling profile and vascular network formation in mSVF-based cultures. mSVF functionality was assessed by analyzing cytokine secretion and vascular network formation in 3D fibrin models. **(A)** Cytokine and growth factor concentrations in mSVF culture supernatants, resuspended in EGM-2 (EG) or DMEM (D) measured by Luminex assay, including IL-10, NGF-b, BDNF, PDGF-BB, VEGF-A, HGF, TNF-α, IL-1β, IL-6, IL-8, and MCP-1 shown as mean ± SD (n = 9). **(B)** Immunofluorescent staining of YFP-HUVECs (green) in coculture with mSVF, eSVF and YFP-HUVECs only (ctrl)cultured in three-dimensional fibrin matrix on day 14, showing CD31-AF647 (white) and Hoechst nuclear counterstain (blue); scale bar = 100 µm. **(C)** Immunofluorescent staining of mSVF cultured in a three-dimensional fibrin matrix on day 14, showing CD31-AF488 (green), CD90-APC (red), and Hoechst (blue), presented as overlay and individual channels; scale bar = 177 µm. Cytokine quantification was performed on 9 independent mSVF isolations.

To evaluate functionality, paracrine activity for relevant effector molecules and the capacity for vascular network formation and support *in vitro* were assessed in mSVF. We characterized the secretome of freshly isolated mSVF for HGF (regenerative/anti-inflammatory/angiogenic), IL-10 (anti-inflammatory), NGF-b and BDNF (neuroregenerative), TNF-a, IL-1b, IL-8 (inflammatory), IL-6 (immunoregulatory), MCP-1 (immunoregulatory/angiogenic) PDGF-BB and VEGF-A (angiogenic). Freshly isolated mSVF secreted neglectable levels of IL-1b, TNF-a, BDNF, PDGF-BB (which were therefore not considered in consecutive studies), low levels of IL-10 and NGF-b, and high levels of VEGF-A, HGF, MCP-1, IL-6 and IL-8 (Figure 3A).

To test the proangiogenic properties of mSVF, a 3D coculture with HUVECs, representing primary endothelial cells with the ability to form vascular networks, was performed. In these cocultures, HUVECs formed distinct vascular network–like structures in the presence of mSVF (Figure 3B), while co-culture with eSVF resulted in more delicate vascular network-like structures. No structure formation was observed in HUVECs cultures alone (ctrl). These results suggest a strong paracrine proangiogenic activity and a pericyte-like, supportive function of cells within the mSVF.

To evaluate the intrinsic network-formation of preserved mSVF microvascular fragments, it was embedded in 3D fibrin monocultures. Within these cultures, three-dimensional interconnected organization of CD31-positive and CD90-positive cells were observed, forming continuous multicellular structures (Figure 3C shows a representative image of CD31-positive and CD90-positive vascular networks). These findings demonstrate that mSVF contains viable cellular components capable of supporting or participating in vascular network organization.

### 3.3 SVF processing results in cell loss in general, filtration additionally alters composition

We investigated the impact of the commonly applied processing steps - washing (resuspension and centrifugation), erythrocyte lysis and filtration - on the number, composition (ASC content, tissue/ECM fragments), and biological properties of the mSVF. Notably, each of these processing steps involves at least one additional centrifugation step.

**FIGURE 4:**
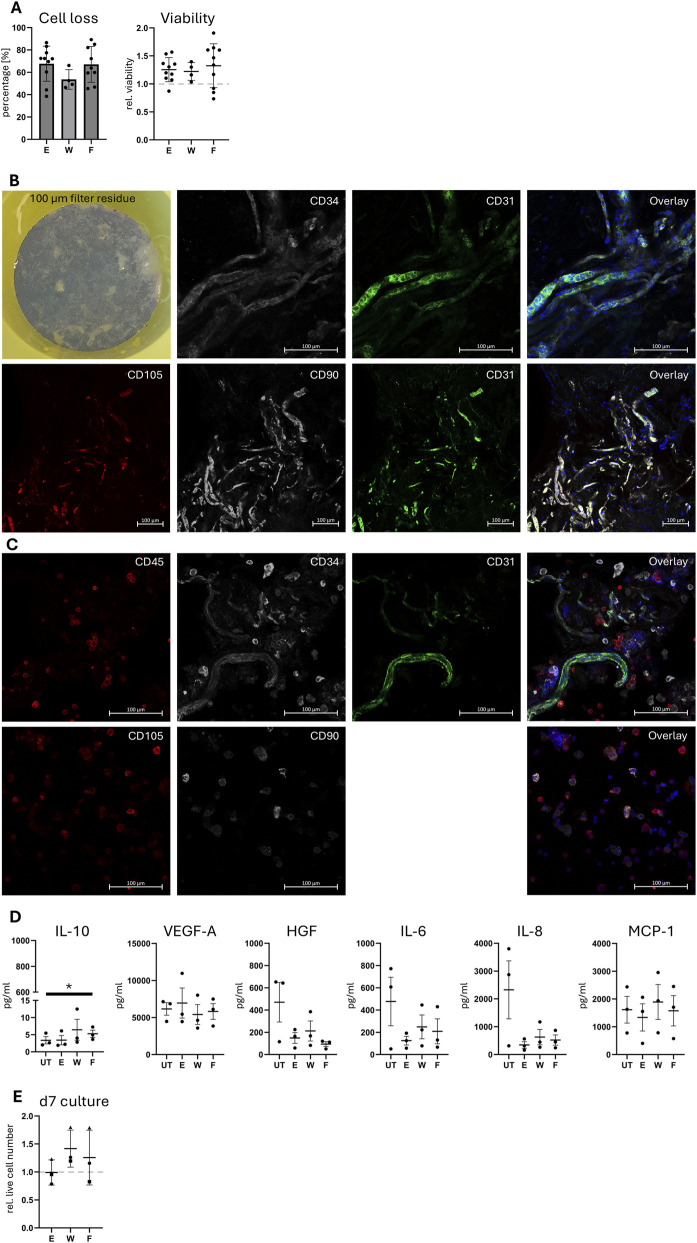
Effects of manipulation steps on mSVF cell retention, phenotype, and secretory profile. Freshly isolated mSVF (untreated: UT) was subjected to the commonly applied processing steps: erythrocyte lysis (E), washing (W) and 100 µm filtration (F), to assess their impact on cell yield, phenotype, and function. **(A)** Total cell loss during the manipulation steps and viability across the different processing conditions is shown relative to its unprocessed state; n: E = 10; W = 4; F = 9. **(B,C)** Representative immunofluorescent images of filter-retained material **(B)** and filtered mSVF **(C)** stained for CD31-AF488 (green), CD105-PE (red) and CD34-APC (white) or CD90-APC (white), with nuclei counterstain Hoechst (blue), presented as overlays and individual channels; scale bar = 100 µm. **(D)** Bioactive factor concentrations of processed mSVF culture supernatants measured by Luminex assay, including IL-10, VEGF, HGF, IL-6, IL-8, and MCP-1; n = 3. **(E)** ASC numbers relative to UT after 7 days of culture, quantified using an automated cell counter across the different manipulation steps; n = 3. All data shown as mean ± SD; p < 0.05 = *. Manipulation-step analyses were performed on erythrocyte lysis (n = 10), washing (n = 4), and filtration (n = 9); Luminex and ASC proliferation assays were performed on n = 3 independent mSVF donors.

Erylysis (E) and filtration (F) resulted in 68% ± 16% and 67% ± 16% cell loss, respectively. Given that washing (W) (resuspension of pellet followed by centrifugation) led to a 54% ± 9% reduction in cell number, these findings indicate that centrifugation accounts for the majority of observed cell loss. Despite the substantial reduction in cell numbers, viability increased across all conditions, reaching a 1.3-fold increase ± 0.2 after erylysis, 1.2-fold increase ± 0.2 after washing, and 1.3-fold increase ± 0.4 after filtration (Figure 4A).

All processing steps were associated with the loss of regeneratively relevant cells. While erylysis and washing preserved both single cells and larger aggregates including microvascular fragments (Supplementary Figures S4A,B), filtration in particular resulted in loss of microvascular and matrix-associated structures. The matrix-associated vascular-like multicellular structures retained on the filter remained positive for CD31, CD34, CD90 and CD105 (Figure 4B). In contrast, the mSVF filtrate predominantly contained single cells along with small microvascular structures. Immunostaining demonstrated that the filtered cells exhibit a regenerative phenotype (positive for CD31, CD34, CD90 and CD105) together with markers of the hematopoietic lineage (CD45 positive) (Figure 4C).

The remaining cell population after processing continued to exhibit pronounced paracrine activity (Figure 4D). While VEGF-A and MCP-1 secretion remained largely unaltered, IL-10 was increased, and IL-6, IL-8 and HGF were tendentially decreased after processing. Especially after filtration, IL-10 levels were significantly elevated (p < 0.03).

Number of ASC in mSVF cultures after 7 days was not affected by erythrocyte lysis, and was slightly increased following washing or filtration, suggesting that the relative ASC content was maintained or enhanced by the processing steps (Figure 4E; Supplementary Figure S4C).

### 3.4 Cryopreservation leads to cell loss but retains functional properties

**FIGURE 5:**
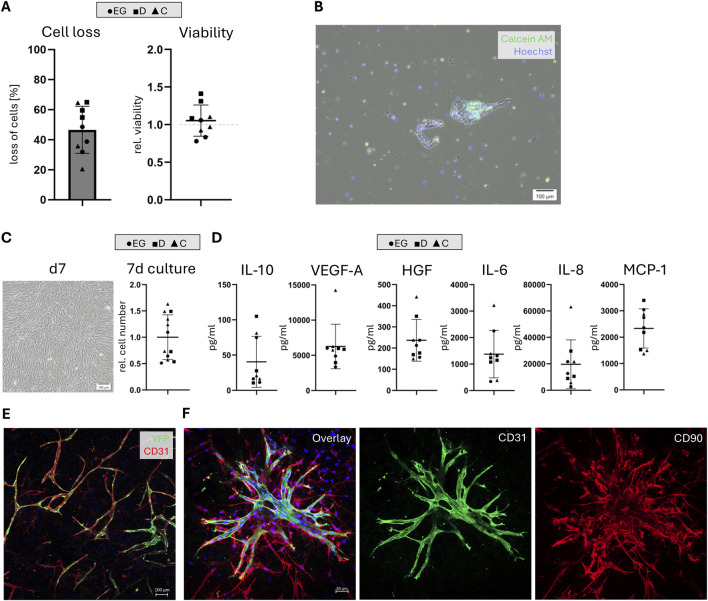
Effects of different cryopreservation media on mSVF cell retention, phenotype, and secretome. mSVF was cryopreserved using different media (EGM2 + 10% DMSO = EG, DMEM + 10% FCS + 10% DMSO = D, and CryoSFM = C) to assess their impact on cell yield, viability, post-thaw proliferation, and functional secretome. **(A)** Percentage of total cell loss across cryopreservation, shown together with relative viability for each condition; n = 9. **(B)** Fluorescence microscopy of non-adherent day 2 mSVF stained with Calcein AM (green) and Hoechst (blue), presented as merged channels with phase-contrast overlay; scale bar = 100 µm. **(C)** Representative phase-contrast image of day 7 ASC cultures showing confluence, alongside relative ASC numbers on day 7 quantified using an automated cell counter. **(D)** Secretome concentrations of cryopreserved SVF culture supernatants quantified by Luminex assay, including IL-10, VEGF-A, HGF, IL-6, IL-8, and MCP-1; n = 9. **(E)** Immunofluorescent staining of YFP-HUVECs (green) in coculture with cryopreserved mSVF cultured in three-dimensional fibrin matrix on day 14, showing CD31-AF647 (red) and Hoechst nuclear counterstain (blue); scale bar = 100 µm. **(F)** Immunofluorescent staining of cryopreserved mSVF cultured in 3D fibrin matrix on day 14, showing CD31-AF488 (green), CD90-PE (red) and Hoechst (blue) in overlay and individual channels; scale bar = 50 µm. All data are represented as mean ± SD. Cryopreservation experiments were performed on n = 3 independent mSVF donors.

Cryopreservation is frequently required in clinical settings to enable multiple treatments. We therefore evaluated the impact of DMSO-based cryopreservation on cell numbers and functional properties of freshly isolated mSVF. The combined process of cryopreservation and subsequent centrifugation for DMSO removal after thawing resulted in a total cell loss 47% ± 16%, independent of the FCS concentrations used in the cryostorage media. Despite this reduction, no relative decrease in cell viability was observed (Figure 5A). To assess the viability of matrix-associated cells, freshly thawed mSVF was stained with Calcein AM and Hoechst. Positive Calcein AM staining in both, single and matrix-associated cell populations, indicated that both remained viable following cryopreservation (Figure 5B).

Cryopreserved mSVF was cultured under standard 2D conditions. Day 7 ASC showed typical spindle shaped morphology independent of the cryostorage media (Supplementary Figure S5) and ASC numbers at day 7 ranged from decrease (to 0.51-fold) to increase (to 1.63-fold) relative to the corresponding, freshly isolated mSVF, reflecting high donor-dependent variability (Figure 5C).

In terms of functional parameters, cryopreserved mSVF maintained its paracrine activity as demonstrated for the selected analytes (IL-10, VEGF-A, HGF, IL-6, IL-8, MCP-1 (Figure 5D). In addition, cryopreserved mSVF retained its ability to support or participate in vascular network organization *in vitro* (see Figures 5E,F).

Using an alternative protocol, simulating clinical application of cryopreserved cells without prior centrifugation for DMSO removal was associated with a reduced loss (17% ± 14%) but decreased viability of 0.77-fold ±0.1 (Supplementary Figure S6A). This approach also showed a tendency towards reduced ASC numbers in culture, with a 0.62-fold ±0.22 decrease relative to freshly cultured mSVF and a 0.70-fold ±0.28 decrease relative to cryopreserved mSVF subjected to DMSO removal (Supplementary Figures S6B,C).

**FIGURE 6:**
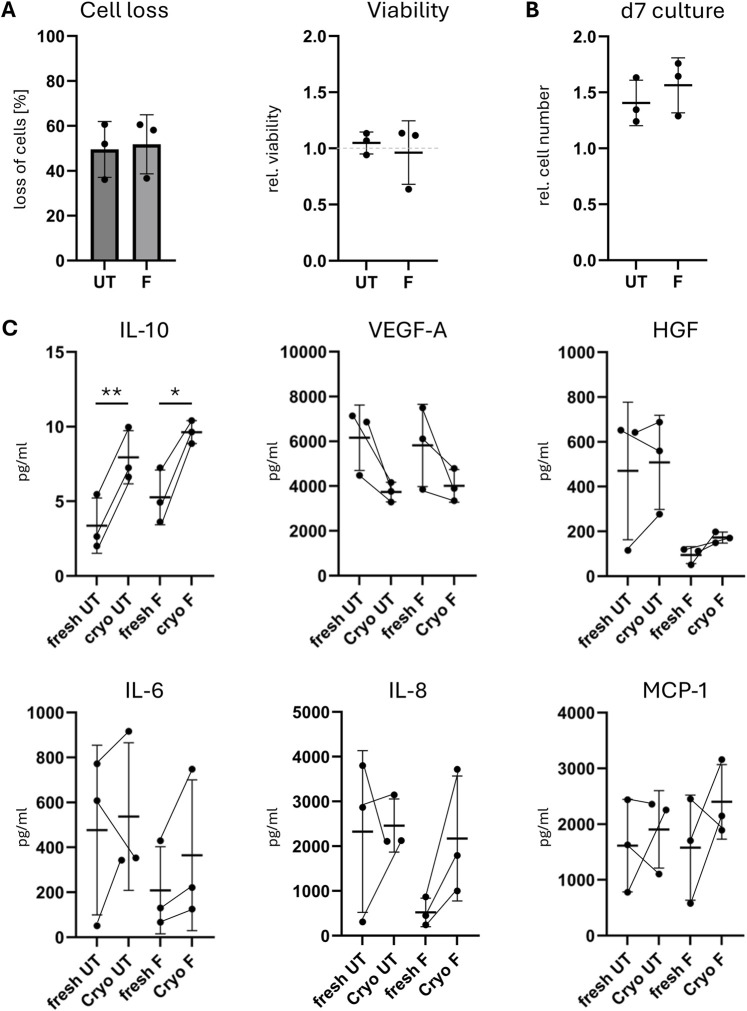
Effects of cryopreservation on filtered mSVF on cell number and secretory profile. mSVF was subjected to 100 µm filtration and subsequent cryopreservation to evaluate their combined impact on cell retention, post-thaw proliferation, and cytokine secretion. **(A)** Percentage of total cell loss and relative viability of untreated (UT) and 100 µm-filtered (F) mSVF following cryopreservation. **(B)** Relative ASC numbers on day 7 quantified using the NC200 cytometer, comparing untreated mSVF to 100 µm-filtered mSVF after cryopreservation. **(C)** Bioactive factor secretion measured by Luminex in untreated (UT) and filtered (F) mSVF before (fresh) and after cryopreservation (cryo), including IL-10, VEGF-A, HGF, IL-6, IL-8, and MCP-1. All data are represented as mean ± SD; p < 0.05 = * and p < 0.01 = **. All quantitative analyses in this figure were performed on n = 3 independent mSVF donors.

In clinical workflows requiring single cell preparations, filtration is an essential step. When combined with subsequent cryopreservation, data obtained from three donors demonstrated comparable cell loss and no marked reduction in cell viability after cryopreservation compared with unfiltered mSVF (Figure 6A). Under both conditions, day 7 culture ASC cell counts were modestly increased following cryopreservation compared with ASC derived from freshly isolated mSVF, supporting the assumption that the relative proportion of ASC is preserved or potentially enriched during the cryopreservation process (Figure 6B).

Bioactive factor secretion (IL-10, VEGF-A, HGF, IL-6, IL-8, MCP-1) was maintained after cryopreservation for unfiltered and filtered mSVF (Figure 6C). For both conditions, IL-10 levels were significantly increased (p < 0.001 for untreated, p < 0.05 for filtered) and VEGF-A levels show a trend to decrease. Therefore, these results indicate that cryopreservation has no further impact on the secretome of filtered mSVF cells.

**FIGURE 7:**
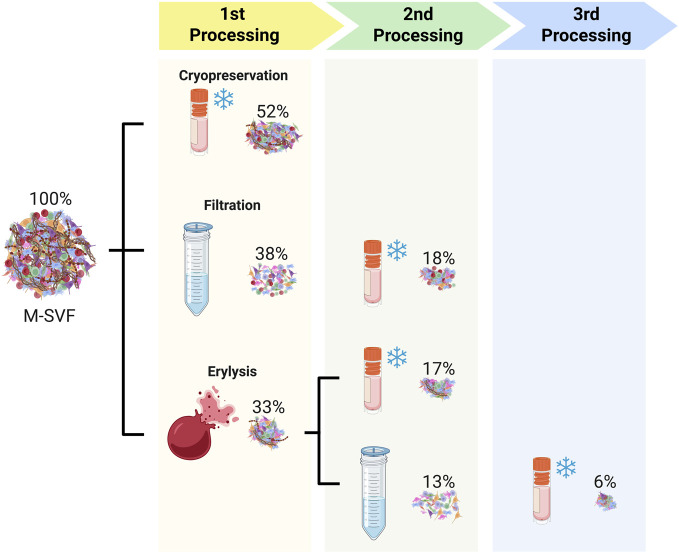
The cumulative effect of processing on mSVF cell. This illustration depicts the theoretical yield of cells available for patient application following the processing steps: cryopreservation, filtration and erylysis, or combinations thereof used in clinical settings. Created with BioRender.com.

In summary, each additional processing step contributes cumulatively to substantial cell loss, resulting in a markedly reduced quantitative cell yield in a potential final product (Figure 7).

## 4 Discussion

In this study we describe an isolation protocol utilizing mechanical tissue homogenization combined with a centrifugation step to generate a mSVF pellet. This cellular composite was characterized immediately after isolation, further processed as indicated, and cryopreserved with or without prior filtration.

The obtained mSVF isolated with the mechanical device in this study is devoid of mature adipocytes and consists of single cells, cellular aggregates, ECM components, and microvascular fragments. Overall, these mSVF cells exhibit a distinct positive expression of mesenchymal stem cell/progenitor markers, such as CD105, CD90 and CD34, which are characteristic of progenitor cell populations, as well as endothelial-associated markers including CD31 (PECAM-1), indicative of endothelial cells, all of which were also reported at varying quantities for SVF obtained by other isolation protocols (Liu et al., 2022).

We obtained a mean total cell yield of 3.4 × 10^5^ cells per ml of lipoaspirate, with an average viability of 38%. Comparative analyses of up to 25 mechanical isolation systems reported a wide range of total cell concentrations, spanning from 0.3 × 10^4^ to 26.7 × 10^5^ total cells/mL lipoaspirate. Notably, cell viability varied considerably between studies, ranging from 45.5% to 95.0%, which was strongly dependent on the specific isolation technique applied and the corresponding target product (fragmented fat from the floating tissue layer vs. SVF cell pellet) (Sforza et al., 2025; Uguten et al., 2024). While some publications report higher cell yields and viabilities, these outcomes were often found for SVF within homogenized fat fragments (Sesé et al., 2019; Trivisonno et al., 2019), with downstream enzymatic digestion for quantitative analysis. In contrast emulsifying mechanical isolation protocols (syringes) (Chaput et al., 2016) are found in lower SVF viability range, comparable with our findings. Part of the lower viability signal after mechanical isolation could also reflect a temporary damage to cell membranes by shear forces, which allows dye flux. However, this hypothesis needs further investigations.

Since in our mSVF product cells remain partially organized in their native microenvironment, including microvascular fragments and multicellular aggregates, these are not detected by conventional cell counting systems, which cannot adequately capture cells within larger three-dimensional structures. Consequently, the total number of viable cells present in the mSVF product is likely underestimated. This is further supported by Calcein AM staining which indicates a high proportion of viable cells within these structures. Despite these limitations, to our knowledge, the aggregate cell counter still represents the best currently available option for heterogenous SVF quality control.

More complex and heterogenous mechanically derived cell therapeutics are often subjected to additional enzymatic digestion before quantitative analysis of cellular composition (Greenwood et al., 2022; Vezzani et al., 2018). When performing additional enzymatic treatment on our cell isolate, we find increased (approximately doubled) viability. While use of enzyme facilitates quantification, the cell material after the additional treatment does not reflect the native properties of the material, thereby limiting its validity for direct comparison between protocols. Further, it does not represent a typical transplant material used in clinics. Whether the observed relatively higher cell death would be detrimental or potentially exert immunomodulatory activity will need to be clarified and will depend–among other aspects–on the clinical setting, the mode of application and the cell dose.

While these larger fragments add to the heterogeneity of the mSVF, they may be especially valuable in the therapeutic setting. Previous studies indicate that cells embedded within ECM retained in a more physiological microenvironment may exhibit improved engraftment potential (Dovgan et al., 2021). The ECM appears to exert a protective effect on cells, supporting their retention, viability and function long-term (“cellular-niche”), whereas complete dissociation into single-cell suspensions may be less favorable for clinical outcomes (Nie et al., 2016). Although mechanical isolation may yield numerically lower cell counts, presence of native ECM as well as tissue fragments may be beneficial for successful engraftment.

Besides this, the therapeutic effects demonstrated by SVF have also been attributed to the presence of ASC or to the crosstalk of several populations in the cell isolate, such as ASC with immune cells, or with endothelial cells to form new vessels.

In this study, the ASC population derived by 2D *in vitro* expansion performed equally with both isolation methods in terms of adhesion and proliferation. Also *in vitro* differentiation capacity towards the adipogenic and osteogenic lineage is comparable, confirming their mesenchymal identity. While others see a reduction in ASC proliferation due to higher shear forces (Chen et al., 2020), or an increase in stemness status due to mechanical forces (Lo Furno et al., 2017) we did not observe altered ASC properties *in vitro*.

Although viable matrix-associated cells did not attach and proliferate under standard monolayer conditions, they represent a biologically active component of the mSVF. Together with adherent single cells, they likely exert significant paracrine activity through the secretion of cytokines, growth factors, and extracellular vesicles (Bollini et al., 2013). From a clinical and surgical perspective, these viable, structurally preserved cell–matrix complexes therefore constitute an important functional element beyond merely plastic-adherent ASC populations.

Our data demonstrate that fresh mSVF possesses strong proangiogenic support in 3D co-cultures with HUVECs, as well as intrinsic network-forming capacity. In addition, key proangiogenic factors, including PDGF-BB, VEGF-A, HGF and IL-8, were found to be secreted from mechanically processed lipoaspirate. This was also reported by others (Copcu and Oztan, 2020; Greenwood et al., 2022; Vezzani et al., 2018), which further underscores the proangiogenic potential of mSVF. It further supports the postulated perivascular origin of proregenerative ASC within the SVF of adipose tissue (Crisan et al., 2008).

For clinical application SVF products are frequently subjected to additional processing steps prior to use or cryopreservation, particularly when intended for repetitive treatments. These steps commonly include centrifugation, washing procedures, erythrocyte lysis, and filtration, all of which are considered essential to obtain a standardized and clinically applicable cell suspension.

Centrifugation represents a key step in SVF isolation, enabling efficient enrichment of desired cells fraction while separating adipocytes and free oil. Although high centrifugal forces are applied for initial SVF pelleting (e.g., 1,000 × *g* in our protocol), previous studies have demonstrated that high centrifugal forces do not adversely affect SVF cell viability, even at forces of up to 4,200 × g (Kurita et al., 2008). Subsequent processing steps are often performed at lower g-forces, to minimize cellular stress. However, our data indicate that these additional centrifugation steps are associated with major progressive cell loss. Furthermore, this was independent of g-force, since increasing the centrifugation speed did not improve overall yield in our hands (Supplementary Figure S7).

Another frequently applied processing step is erylysis to minimize the number of erythrocytes administered. Its impact in autologous transplant material remains controversial, as both lysis byproducts (Aguilo-Seara et al., 2023) and intact erythrocytes may impair mesenchymal and progenitor cell function (Assmus et al., 2010). In addition, erythrocytes have been shown to bind and act as reservoirs for inflammatory cytokines such as IL-8 or MCP-1, thereby modulating their bioavailability (Jiménez-Sousa et al., 2019). In our study, erylysis reduced total nucleated cell numbers without significantly affecting paracrine secretion and ASC function.

Filtration is commonly used in ASC isolation to generate a more homogeneous, smaller-particle suspension that facilitates standardized and uniform material application and enables analysis by conventional cell counting and flow cytometry. However, such analytical “gold standards” inherently require additional processing steps, including centrifugation, washing, and filtration or enzymatic dispersion, all of which can substantially affect cell yield. Our data demonstrate that filtration leads to a loss of both single cells and, in particular, larger microvascular fragments, which are increasingly recognized for their potential benefit given their structural integrity and regenerative activity (Gao et al., 2025; Nalbach et al., 2021). While filtered mSVF retains ASC, immune cells, and small vascular aggregates, it is accompanied by alterations in the secretory profile. Notably, IL-10 secretion was significantly increased in filtered mSVF, likely reflecting contributions from anti-inflammatory immune cells and ASC (Choudhery et al., 2025). In contrast, HGF, a key anti-inflammatory and anti-apoptotic mediator previously reported to be enriched in SVF compared to isolated ASC cultures (El-Habta et al., 2018), was reduced after filtration. These findings suggest that multicellular structures such as microvascular fragments contribute substantially to the regenerative secretome. While filtration reduces heterogeneity and facilitates standardized analysis, it may in turn compromise aspects of the therapeutic potential by depleting structurally and functionally relevant cell–matrix assemblies.

In clinical settings, SVF cells can either be administered freshly for regenerative or cosmetic indications or cryopreserved to facilitate repeated administrations, (limiting the number of liposuctions). Moreover, cryopreservation enables comprehensive quality control of the cellular product prior to use, including sterility testing, characterization of cell populations, and evaluation of the proliferative capacity of ASC, thereby supporting safe and quality-controlled clinical application.

Optimal preservation remains critical for maintaining regenerative potential. Various cryoprotective media are available, among which DMSO is widely used despite being associated with potential side effects and cellular alterations. Nevertheless, DMSO has been superior in support for post-thaw proliferation of ASC compared to alternative agents such as trehalose (Favaretto et al., 2023; Zekušić et al., 2025). In the present study, we therefore evaluated three cryoprotective media containing 10% DMSO with varying FCS concentrations including one serum free medium.

In line with the findings of others, we observed an approximate 50% reduction in total cell number following cryopreservation with a washing step, while cell viability remained stable including the microvascular structures. Solodeev et al., showed that after cryopreservation enzymatically isolated SVF preserved its stem cell potency (90% FCS; 10% DMSO) with no significant change in ASC properties *in vitro* (Solodeev et al., 2019). Our data demonstrate, that also mSVF cryopreservation shows unchanged proliferative capacity of ASC, as well as preserved, proangiogenic and immunomodulatory secretory properties and maintained support and participation in vascular network organization in a 3D culture.

In contrast, when assessing the effect of cryopreservation without centrifugation, cell numbers decreased by approximately 20% suggesting that the majority of cell loss may be associated with the washing procedure. However, omission of this step resulted in reduced viability, which was also reflected in the proliferative capacity of ASC, where a reduction of approximately 50% was observed. Based on previous findings that approximately 90% of DMSO is removed during the first washing step after thawing, the remaining presence of DMSO is likely to be responsible for this effect (Decot et al., 2009).

No statistically significant differences were detected between the tested cryopreservation media. From a GMP compatible perspective, this suggests that serum-free media or an autologous serum may be preferable (Agostini et al., 2018), given the lack of additional benefit from FCS supplementation and the potential risks associated with its use.

Taken together, the BMM utilized in this SVF study represents an intermediate processing level among currently used isolation systems. The resulting cell isolate is neither a mechanically fragmented adipose tissue preparation, nor a fully dissociated single-cell suspension, but a heterogeneous mixture of cells within ECM, including preserved microvascular structures. We emphasize that the proregenerative properties of SVF likely result from a complex interplay between diverse cell populations, like ASC or perivascular cells *via* paracrine signaling (Vezzani et al., 2018), ECM components, and microvascular fragments that may act as sprouting units contributing to neovascularization and tissue remodeling (Tremolada et al., 2023). As potential next step, it may be worthwhile investigating the observed effects in i*n vivo* in preclinical settings. Such studies could provide additional insights into the proangiogenic potential of the approach and help to better characterize the dynamic processes involved under physiologically relevant conditions.

However, it is very important to keep in mind, that the SVF, which is administered in a therapeutic setting may not fully reflect the cellular composite that has been quantitatively and qualitatively characterized in the associated quality control, as the required processing steps may selectively deplete or alter potentially beneficial cell populations or extracellular components.

## 5 Conclusion

In conclusion, greater awareness of how each processing step affects cell yield and composition is essential to improve protocol standardization and to enhance comparability across studies and clinical outcomes. Although MSC-based cell therapy has come a long ways since the early clinical application pioneered by Cancedda and others (Quarto et al., 2001), this study highlights that there is still a need for optimization in cell therapy, including the basic processing requirements.

## Data Availability

The raw data supporting the conclusions of this article will be made available by the authors, without undue reservation.
