# Supplementary material for: Impact of processing steps on cellular content of mechanical isolated stromal vascular fraction
Source: Front Bioeng Biotechnol. 2026 Jul 17;14:1869470. doi: 10.3389/fbioe.2026.1869470 (PMC13424472; doi:10.3389/fbioe.2026.1869470)
Supplement: Supplementary file 1 [file Supplementaryfile1.docx]

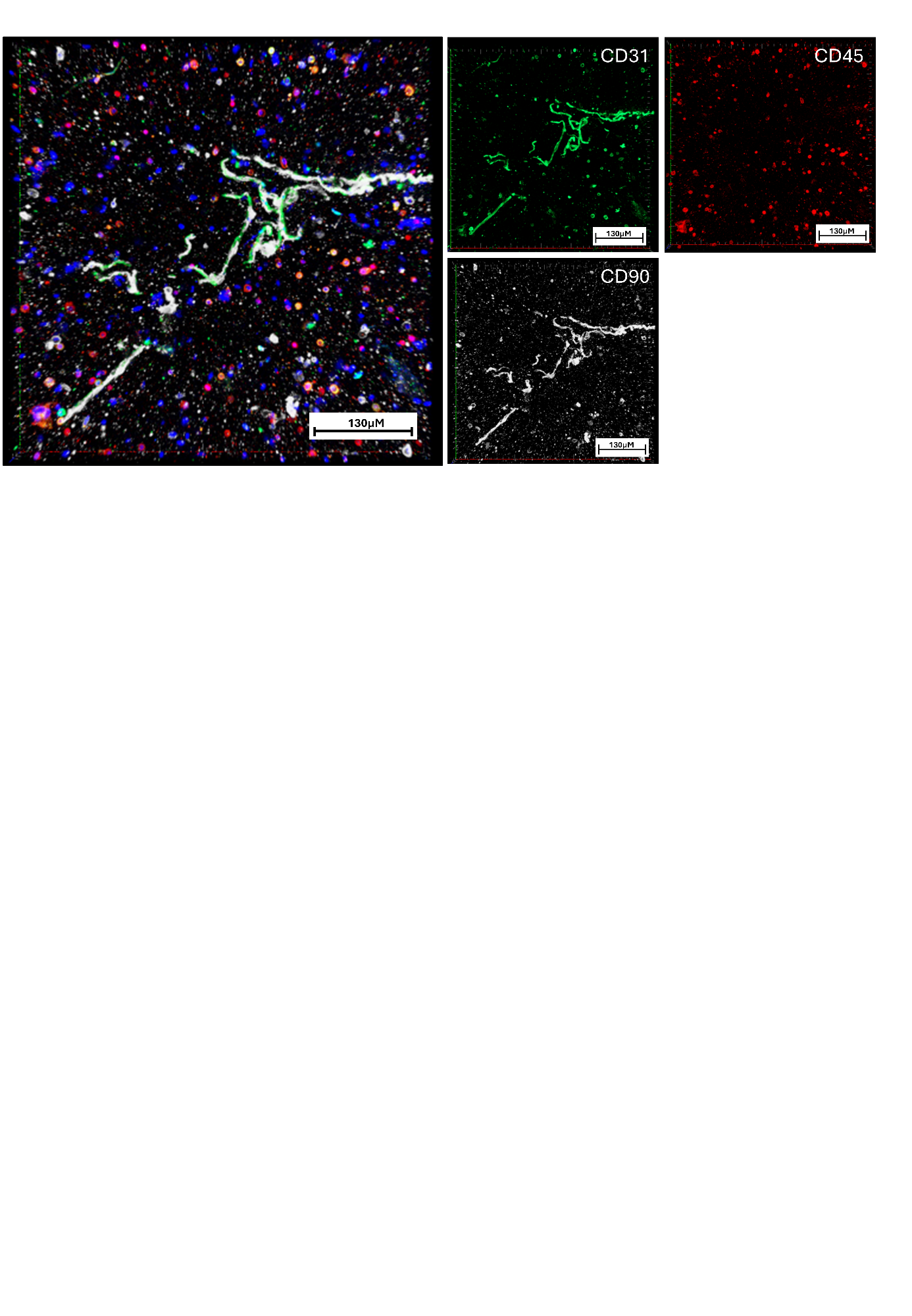


**Supplementary Figure 1. Immunofluorescent characterization of freshly isolated mSVF embedded in a 3D fibrin matrix.** Representative three-dimensional immunofluorescent staining showing CD31‑AF488 (green), CD45‑PE (red), and CD90‑APC (white) with Hoechst counterstain presented as overlays and individual channels; scale bar= 130 µm.


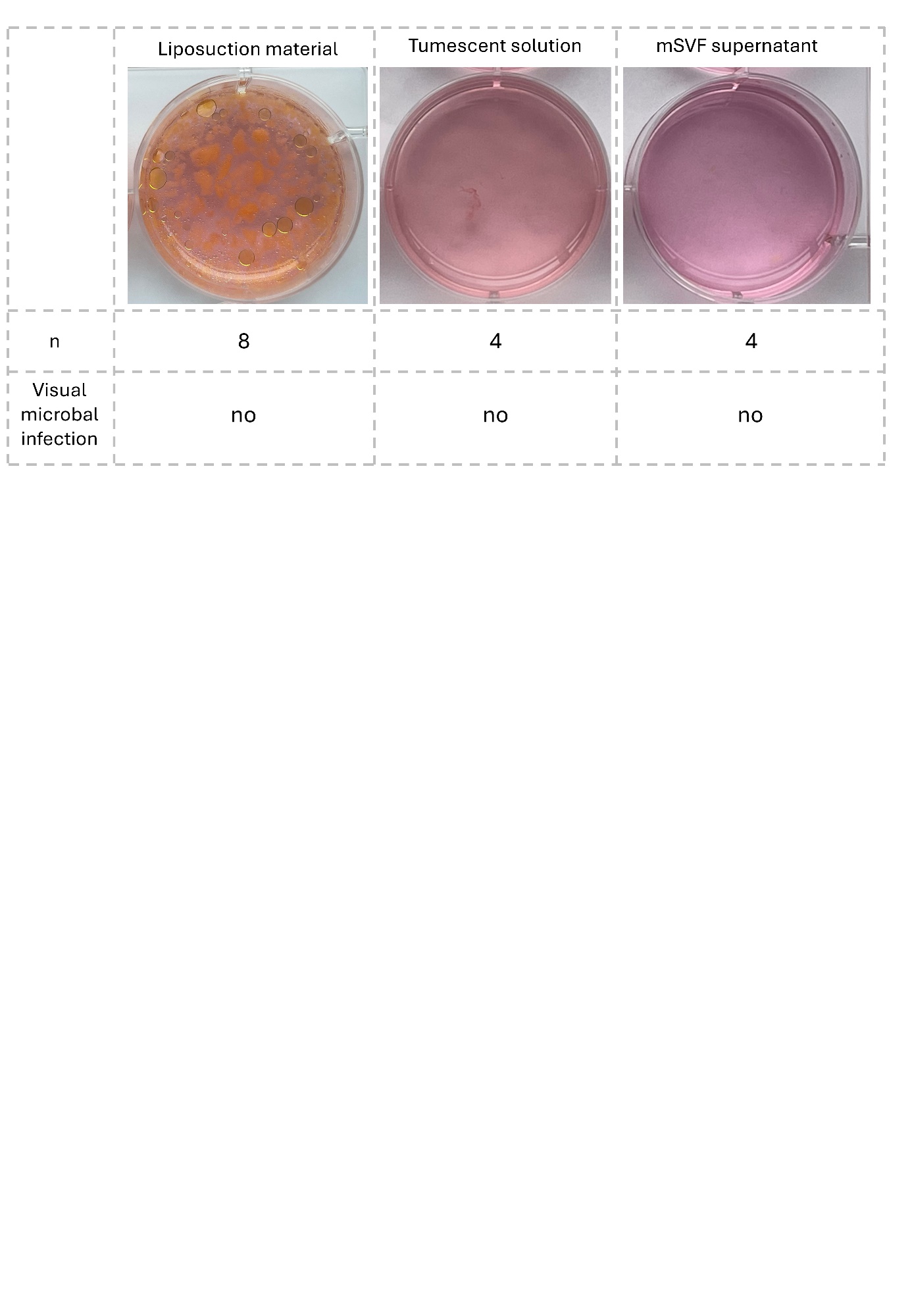
 **Supplementary Figure 2. Sterility assessment of adipose tissue-derived materials.** Representative sterility cultures of lipoaspirate, tumescent solution, and mSVF supernatant samples incubated in DMEM + 10%FCS for a week. All samples remained clear throughout the incubation period, with no visible turbidity, colonies, or other signs of microbial contamination. Sterility assessed across fat (n = 8), tumescent solution (n = 4), and supernatant (n = 4), and all samples tested negative.


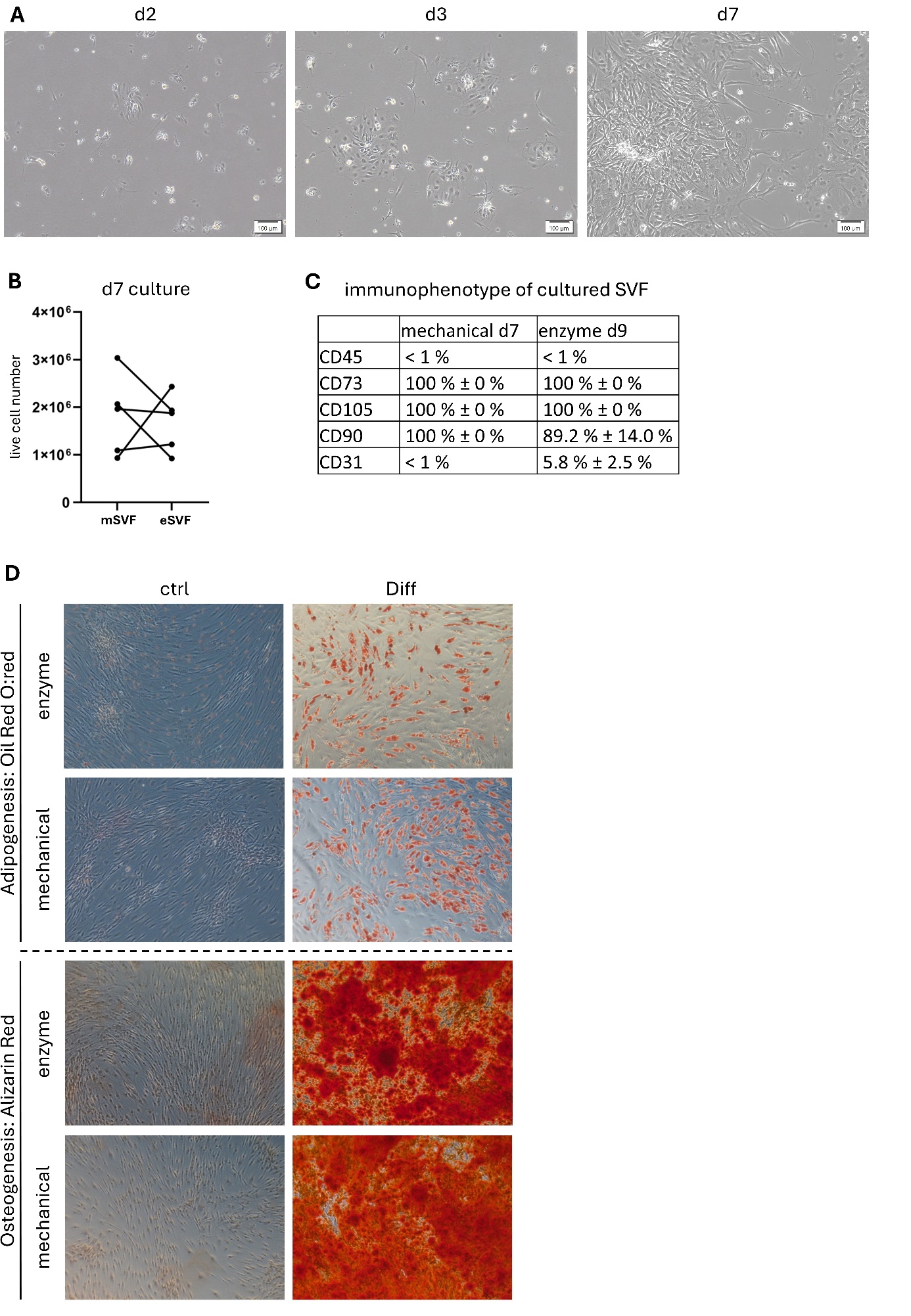


**Supplementary Figure 3.** **ASC proliferation and differentiation capacity in cultures of mechanically (mSVF) and enzymatically isolated SVF (eSVF).** **(A)** Representative phase‑contrast images of eSVF cultures on day 2, day 3 and day 7 showing endothelial cobblestone-like colonies together with spindle-shaped ASCs scale bar = 100 µm. **(B)** Live cell numbers quantified on day 7 using the automated cell counter for eSVF and mSVF. **(C)** Immunophenotype of cultured mSVF (d7) and eSVF (d9) harvested upon confluency and analyzed by flow cytometry for CD45-V500; CD73- FITC; CD105-PE; CD90-APC and CD31-FITC. n=3 for each condition; data are presented as mean ± SD. **(D)** Expanded ASCs were capable of undergoing adipogenic and osteogenic differentiation. Representative images of Oil red O staining (red) of adipo-differentiated and control (non-differentiated) samples and Alizarin Red staining (red) of osteo-differentiated and control samples for eSVF and mSVF.


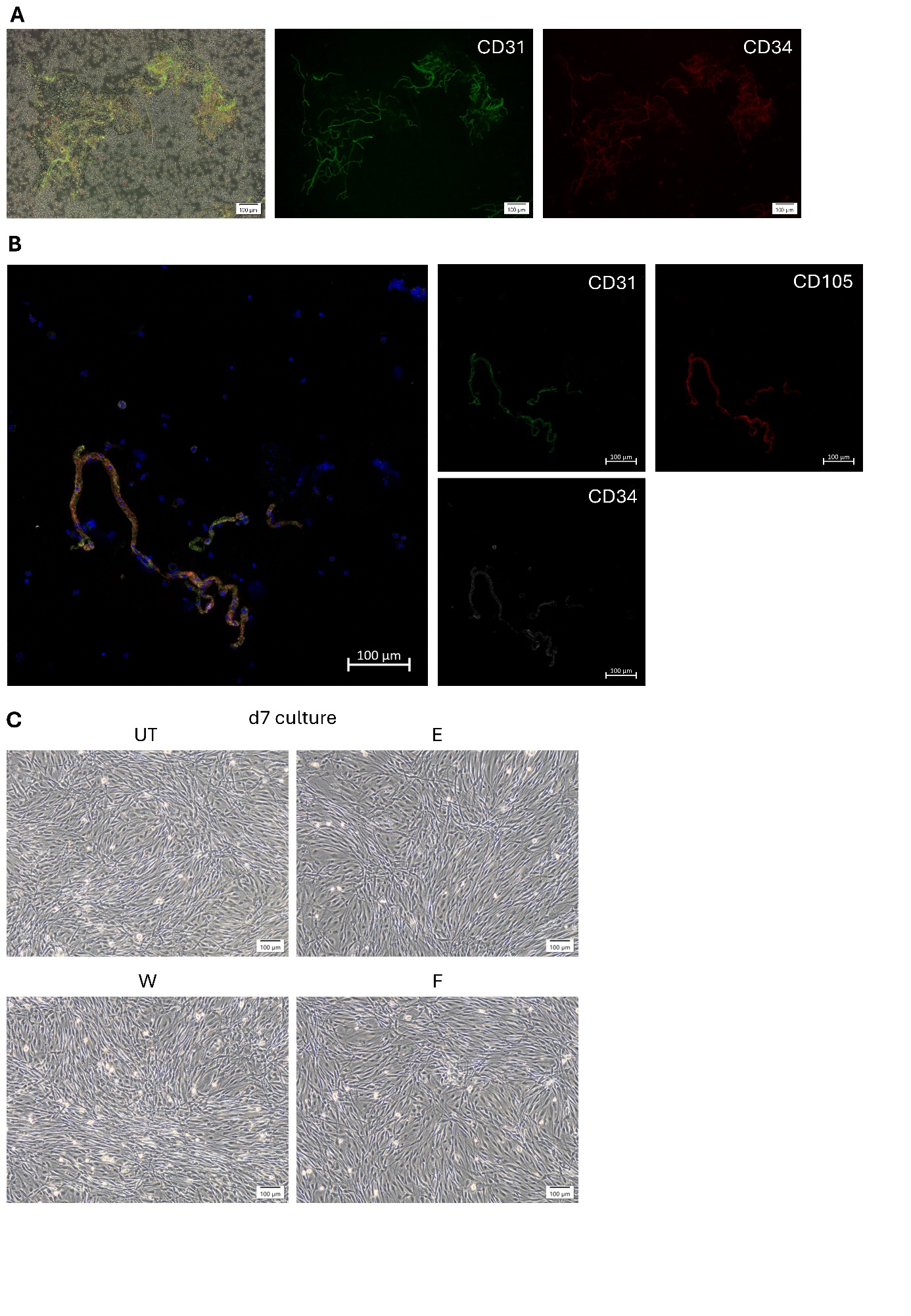


**Supplementary Figure 4: (A)** Representative immunofluorescent stainings of washed mSVF showing CD31‑AF488 (green), CD34-ACP (red), with phase contrast overlay and individual channels; scale bar = 100 µm. **(B)** Representative immunofluorescent stainings of mSVF after erylysis stained for CD31‑AF488 (green), CD105‑PE (red) and CD34‑APC (white), with nuclei counterstain Hoechst (blue), presented as overlays and individual channels; scale bar = 100 µm. **(C)** Representative phase‑contrast images showing ASC confluence on day 7 following different manipulation steps, including untreated (UT), erythrocyte lysis (E), washing (W), and 100 µm filtration (F); scale bar = 100 µm.


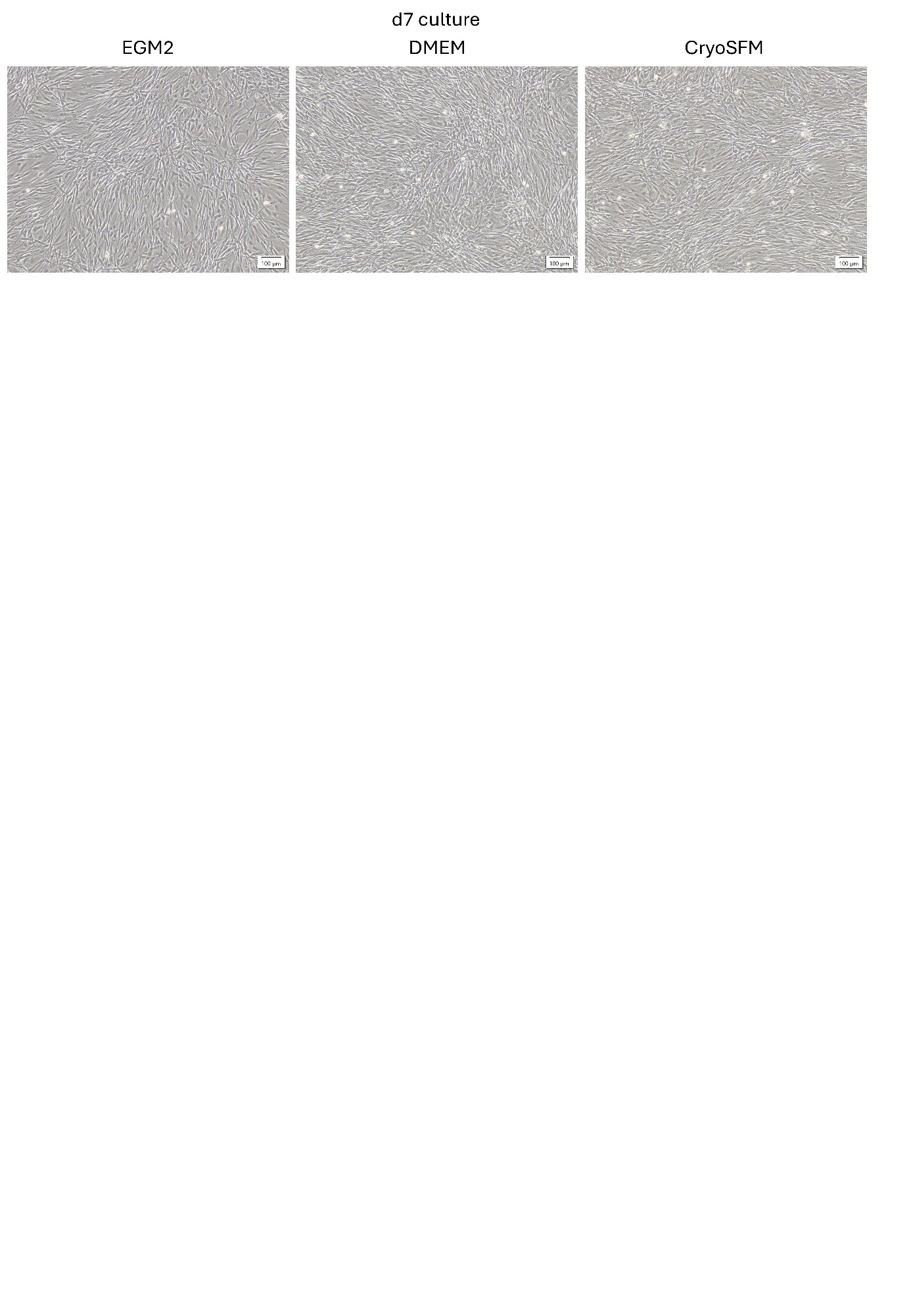
**Supplementary Figure 5: Effects of different cryostorage media on post-thawn ASC proliferation.** mSVF was cryopreserved using different media (EGM2 + 10 % DMSO = EGM2, DMEM + 10 % FCS + 10 % DMSO= DMEM, and CryoSFM). After thawing, samples were centrifuged to remove DMSO and subsequently cultured in EGM2 to evaluate the effect of cryostorage handling and media and on post-thaw proliferation. Representative phase‑contrast images of day‑7 ASC cultures. (Magnification = 10× scale bar = 100 µm)**.**


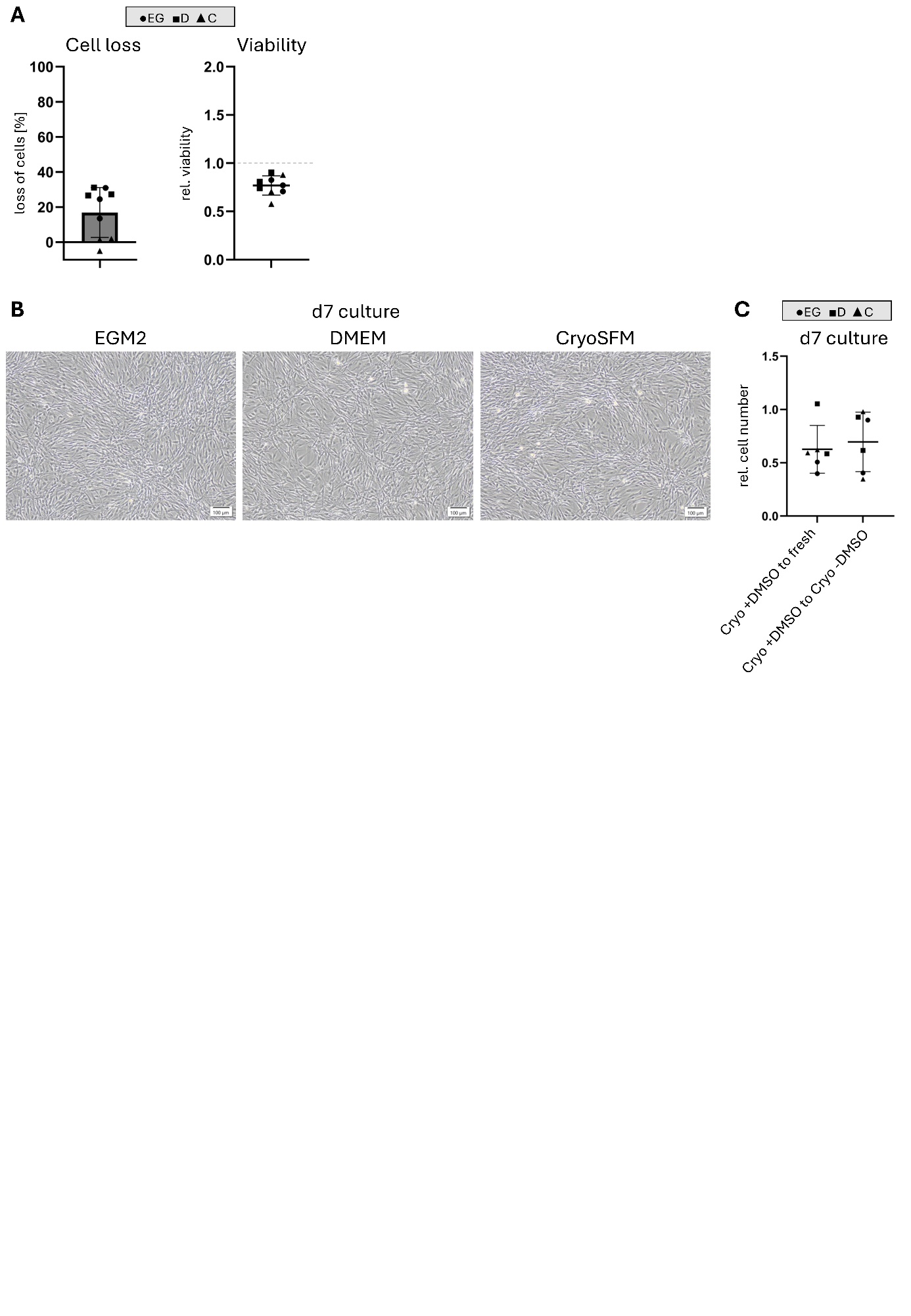


**Supplementary Figure 6**: **Effects of DMSO on mSVF cell retention and viability after cryopreservation.** Cryopreserved mSVF (EGM2 + 10 % DMSO = EG, DMEM + 10 % FCS + 10 % DMSO= D, and CryoSFM= C) was cultured without removing DMSO to evaluate its impact on cell yield and viability. **(A)** Percentage of total cell loss after cryopreservation across the different media conditions, shown together with relative viability for each condition; n=9. **(B)** Representative phase‑contrast images of day‑7 ASC cultures. (Magnification = 10× scale bar = 100 µm). **(C)** Relative ASC numbers on day 7 quantified using an automated cell counter, comparing cultured cryopreserved mSVF (cryo +DMSO) to freshly cultured mSVF (fresh) and to cryopreserved cultured controls (cryo -DMSO); n=6.


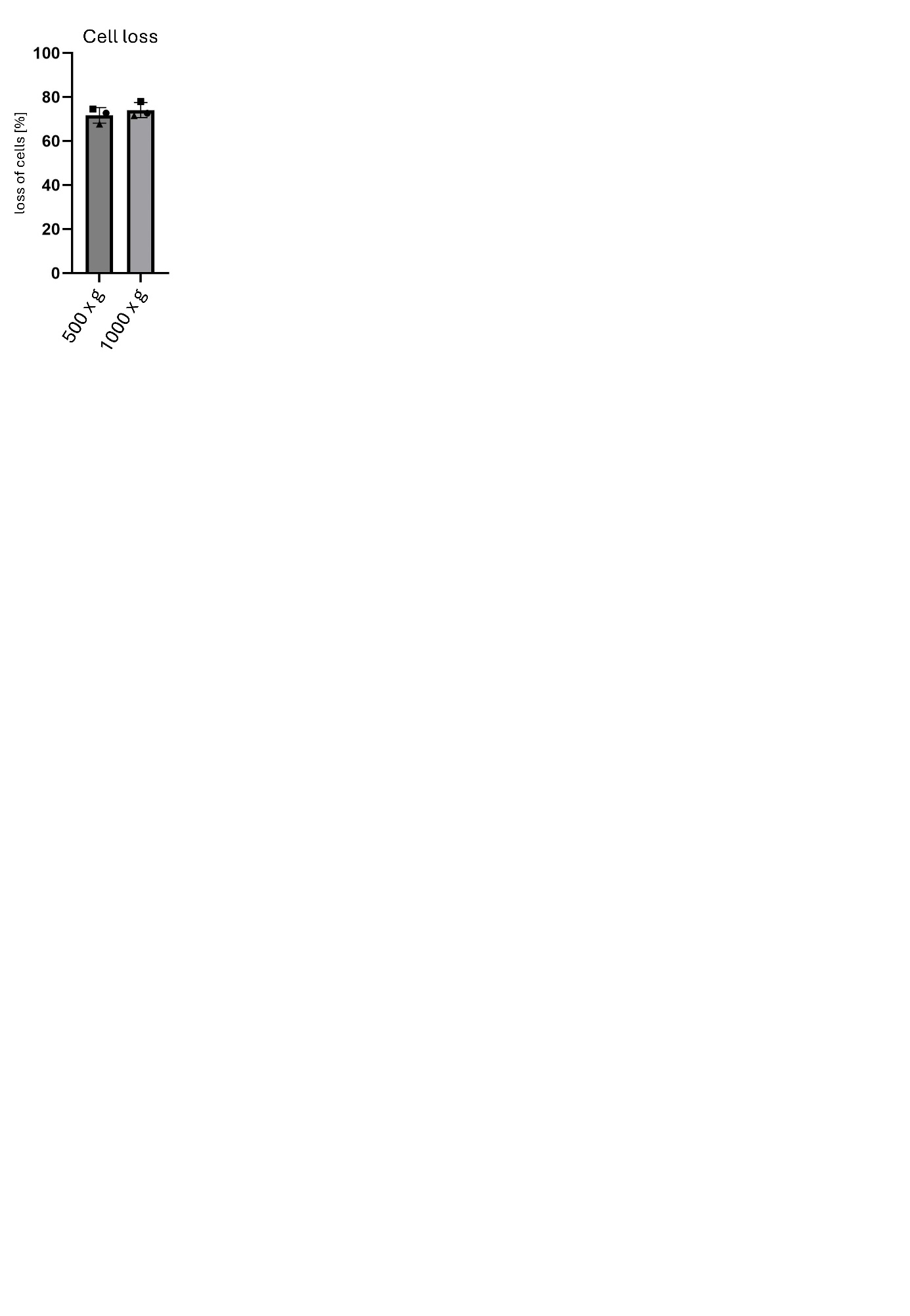


**Supplementary Figure 7**: **Effects of different centrifugation g-forces during washing on cell yield.** SVF pellets were subjected to a washing step using different centrifugation forces (500 × g and 1000 × g) to assess their impact on cell loss. Total cell loss is shown as percentage relative to the unprocessed state; n=3.
